# Supplementary material for: ATAD3 megadalton complex in Plasmodium falciparum is essential for mitochondrial and cellular viability
Source: PLoS Pathog. 2026 Jun 3;22(6):e1014317. doi: 10.1371/journal.ppat.1014317 (PMC13249166; doi:10.1371/journal.ppat.1014317)
Supplement: S1 Fig — (B) Phylogenetic analyses showing the evolutionary relationship between homologs of ATAD3 amongst alveolates and other eukaryotes including Homo sapiens, Arabidopsis thaliana, Caenorhabditis elegans, Plasmodium falciparum, Toxoplasma gondii, and Cryptosporidium parvum. AFG3-like proteins were chosen for the putative outgroup as selected members of the AFG3-like proteins exhibited the highest similarity to PfATAD3 and this analysis is limited to the NTPase domain because no other AAA+ protein subclass has detectable similarity with the ATAD3 domain. (C) Phylogenetic analyses showing the evolutionary relationship between homologs of ATAD3 amongst alveolates and other eukaryotes including Homo sapiens, Arabidopsis thaliana, Caenorhabditis elegans, Plasmodium falciparum, Toxoplasma gondii, and Cryptosporidium parvum using the full-length ATAD3 sequence. (D) Multiple sequence analysis of ATAD3 homologs in apicomplexan parasites. (PDF) [file ppat.1014317.s001.pdf]

A

**Scores**	***Pf*ATAD3**	**Human ATAD3**
pLDDT (average confidence estimate score calculated per atom between 0 and 100)	66.45	68.79
pTM (predicted template matching score between 0 and 1)	0.38	0.38
ipTM (predicted interface template matching score between 0 and 1)	0.35	0.34
ranking score (predictive score between -100 and 1.5 incorporating pTM, ipTM, fraction_disordered, and has_clash scores)	-0.44	0.49

B

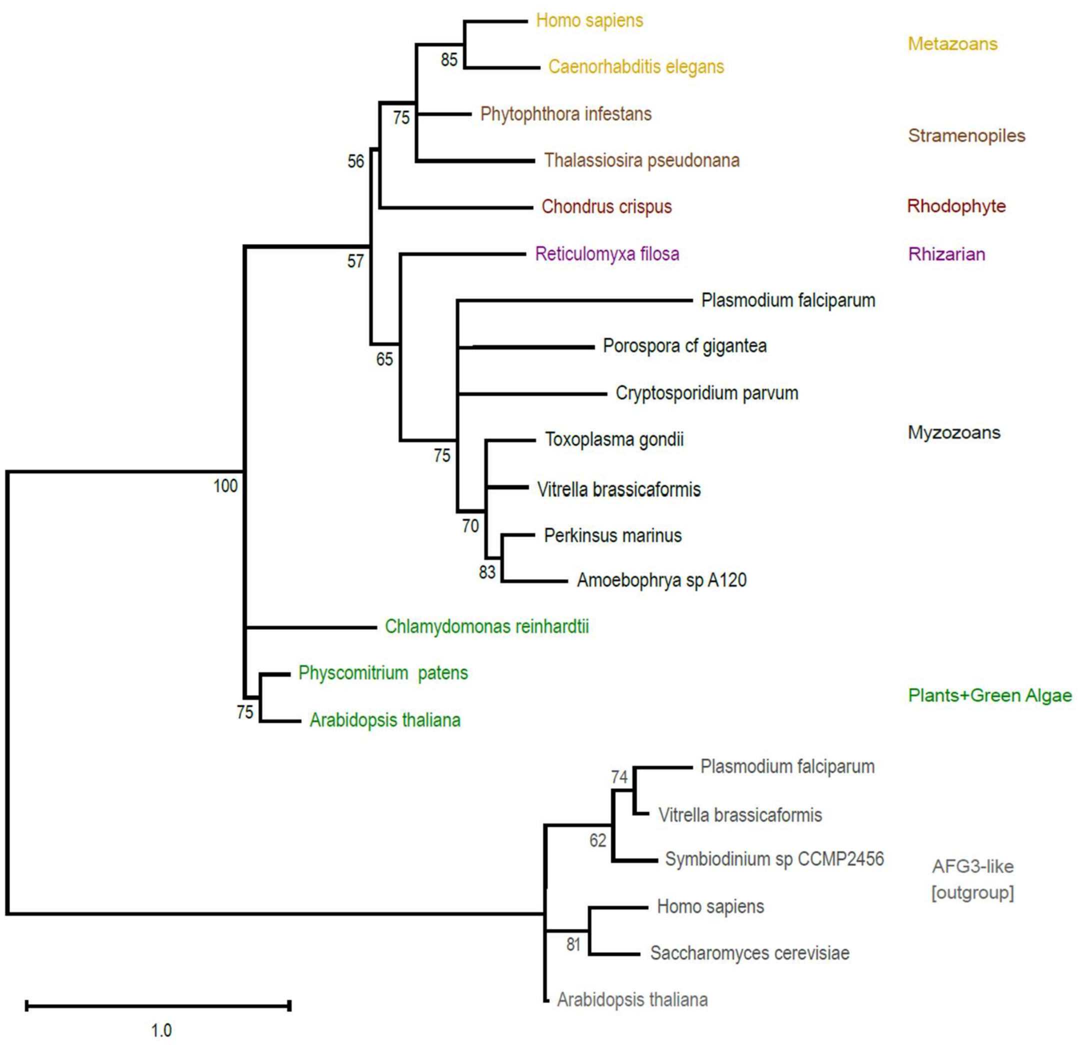

Homo sapiens
Caenorhabditis elegans
Metazoans
85
75
Phytophthora infestans
Thalassiosira pseudonana
Stramenopiles
56
Chondrus crispus
Rhodophyte
57
Reticulomyxa filosa
Rhizarian
Plasmodium falciparum
Porospora cf gigantea
65
Cryptosporidium parvum
Toxoplasma gondii
Myzozoans
75
Vitrella brassicaformis
100
70
Perkinsus marinus
83
Amoebophrya sp A120
Chlamydomonas reinhardtii
Physcomitrium patens
Plants+Green Algae
75
Arabidopsis thaliana
74
Plasmodium falciparum
Vitrella brassicaformis
62
Symbiodinium sp CCMP2456
AFG3-like [outgroup]
Homo sapiens
81
Saccharomyces cerevisiae
Arabidopsis thaliana
1.0

C

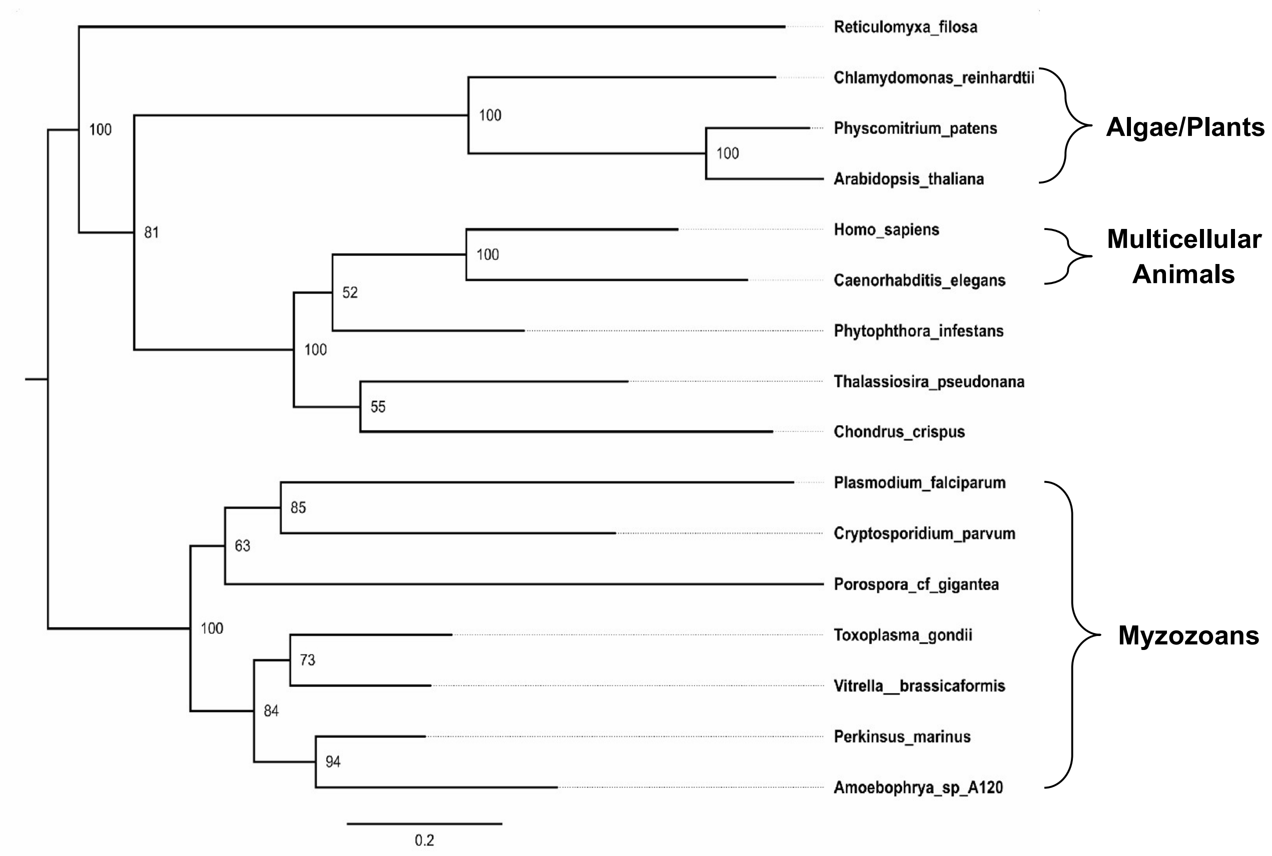

Reticulomyxa_filosa
Chlamydomonas_reinhardtii
Physcomitrium_patens
Arabidopsis_thaliana
Homo_sapiens
Caenorhabditis_elegans
Phytophthora_infestans
Thalassiosira_pseudonana
Chondrus_crispus
Plasmodium_falciparum
Cryptosporidium_parvum
Porospora_cf_gigantea
Toxoplasma_gondii
Vitrella__brassicaformis
Perkinsus_marinus
Amoebophrya_sp_A120
Algae/Plants
Multicellular Animals
Myzozoans
100
100
100
81
100
52
100
55
85
63
100
73
84
94
0.2

D

```
P.falciparum/1-663     1 MNFPNLSKKINSTVSSNKPYSSSDEK--------GGEHITGNFDPTALERGAKALKELDQSSNSSK  58
P.vivax/1-665          1 MNFPNISKKMAAPV-------AGEEK--------GGEHITGNFDPTALERGAKALKELDQSSNSKK  51
T.gondii/1-588         1 MFFPSVGGSGAA----GAAPTLTSQK---VNLPGKDDDITGKFDPTALERGAKALKELDSSPNAAK  59
B.besnoiti/1-577       1 MFFPSVGAGASG----AALPTHKPSA-------GKDEDITGKFDPTALERGAKALKELDSSPNAAK  55
E.tenella/1-595        1 MYFPSFGGSSAVPS--AALPNAIPKK------EDDGESITGKFDPSALERGAKALKELDSSPNAAR  58
E.falciformis/1-597    1 MYFPSFGGGSAVPS--AALPNAIPKK-------EDVDSITGKFDPTALERGAKALKELDSSPNAAR  57
B.bovis/1-567          1 M----FGFGSPQV---PSSAIPPLPN--------DDANITGKFDPTALERGAKALKMLDSSPNAQK  51
T.equi/1-568           1 MAFSGFGFGGGTPG--PAPVQPSSSK--------DDKNITGKFDPTALERGAKALRMLDSSPNAQK  56
T.parva/1-558          1 M--STFGFGRSFP---TPNPAPSSNK--------DDNNITGKFDPTALERGAKALKMLDSSPNSQK  53
B.microti/1-558        1 MFYSSGGSKG------FKNTYKEEDG--------GDGNITGKFDPSALERGANALKALDASPNARM  52
C.muris/1-635          1 MFFRSLGHSS-QNS--GTLSQLKTEK-------NVVDNISGYFDPTALERGAKALKELDASPNAQK  56
C.parvum/1-627         1 MFFSGFGGRQGSPN--PDSGSGDKDSGGKYTGGNGGGSINGNFDPTALERGAKALKQLDSSPNAQK  64

P.falciparum/1-663    59 AFEVIKLQELTKQKEYEKQMEELSLQRAQHMSNRMRMENEEKRKTINYQQEQERITAEYKTKLEAE 124
P.vivax/1-665         52 AFELIKLQELTKQKEYEKQMEELSLQKAQYLSNKMRIENEEKRKTINYQQEQERITAEYKTRLEAE 117
T.gondii/1-588        60 AFEVTKLQEQTKQKQLQKEMEELATVRARAQAEHARAEAEERRKTINHAQEQERVTAQYRAQLEAE 125
B.besnoiti/1-577      56 AFEVTKLQEQTKQKQLQKEMEELAAVRMRAQAEHARTEAEERRKTINHAQEQERVTAQYRAQLEAE 121
E.tenella/1-595       59 AFEVIKLQELSKQKELQKQIEQIAAARAQAQTERTKTEGEERRKTIDHQQEQERVTAQYKAKLEAE 124
E.falciformis/1-597   58 AFEVIKLQEMTKQKELQREMEQISAARAQAQTERTKTEGEERRKTIDHQQEQERITAQYKAKLEAE 123
B.bovis/1-567         52 AFELTKMQEMTRQHEIQKEIQQMQLRQSELGAQRARVESDEKRKLMAQQQEQDRITAQYKAKLEAE 117
T.equi/1-568          57 AFELTRLQELTRQQEIKKEIEQMHLRQTELGAQRARIEGDERRKLAAQQQEQERITAQYKAQLESE 122
T.parva/1-558         54 AFELTKLQEMTKQQELQMQIEQMRLRQGELGTQKAKVESDERKKLLSHQQEQERITAQYKAKLEDE 119
B.microti/1-558       53 AFELTKLQELTRQQEIQREIQQLQLRQSEAVSQKSRIEGEERRKLLSQQQEQERITAQYKAQLEAE 118
C.muris/1-635         57 AFDLVRLQEVTKQMEIEKEMEQSAMYRTQAHNEKVRIEAEERRKTISHQQEEERVTAQYKARLEAE 122
C.parvum/1-627        65 AFEIIKLQEKTKQKELERDIEQSSAYRSKANLERTRIEADERRKTITHQQEEERATSQYKAKLETE 130

P.falciparum/1-663   125 SYQKKLLDQQKQNEEWLRNQHEQYLRQENIRKRNELELMNIKMKQIKEEKRLERENMKARIFEENK 190
P.vivax/1-665        118 AYQKKLLDQQKQNEEWLKNQHEQYLRQENIRKRNELELLNLKMKQIREEKSLERENMKAKIQEENK 183
T.gondii/1-588       126 AYQKKLQDQQKQNEVWLEQQHQQFLRQQELRKRQEQELLEMRRQQMREEKALEREVMRERIQEETK 191
B.besnoiti/1-577     122 AYQKKLQDQQKQNEVWLEQQHQQFLRQQELRKRQEQELLEMRRQQMREEKAMEREIMREKIQEETK 187
E.tenella/1-595      125 AYQKKLQDQQRQNEEWLRQQHKQFLLQEEQRKKTEAEMLEMRRQQLREEKALEEKLQKEKIREEAK 190
E.falciformis/1-597  124 AYQKKLQDQQRQNEEWLRQQHQQFLRQEEQRKKTEAEMLEMRRKQMREEKAMERELQAERIREEAK 189
B.bovis/1-567        118 AYQKKLQDQRRQNEEWLNQQHQQFLRQEEARKKTEMEILNMRKAQIREEKALERENIKARVQEEGR 183
T.equi/1-568         123 AYQKKLIDQRRQNEEWLQQQHQQFLRQEEIRKKTELDILEMRKAQMKEEKALERENIRAKVQEESK 188
T.parva/1-558        120 MYQKKLHDQRKQNEEWLQRQHQQFLKQEEIRKKTETEILNMRKEQMKQEKELERENLVAKVREENM 185
B.microti/1-558      119 AYKNKLQEQKKQNEEWLELQHKQFLKQEELRKKTEMDILKMKKEQAEHEKSLERESIKVKVREEAK 184
C.muris/1-635        123 AYQKKLYDQQEQNTSWLKQQHEQFLLQEKVRKDNEQEILLLRQRQLEEEKRLEKENIKIKIREKTR 188
C.parvum/1-627       131 AYYKKLKEQESQNARMLKQQHDKFLEQEEIRKKNEREILEMKRRQSEYENKLQQENIKVRIREETI 196

P.falciparum/1-663   191 GLIERERKNLDIHLTTLRTKADEDRKTKIESINKYFEQFNNSLFLFLNDKQKLYRFALTITLTSIG 256
P.vivax/1-665        184 GLIERERKNLDIHLKTLRMKADEERKTKLESIGKYFEQFNNSMFLFLNDRERLYRFVLVVTLTSVG 249
T.gondii/1-588       192 GRIKQERENVDIHLREMRAKAAEFRKTRLETLQTVFSGVGNAFNELMSDRSRLATLVGSLSLLACG 257
B.besnoiti/1-577     188 GRIKQERENIDIHLRELRAKAAEYRKTRMDTLHTVFSGVGNAFNELMSDRSRLATLVGSLTALACG 253
E.tenella/1-595      191 GRILQERENIDVHLRAMRARAAEERKTKLESLEKTFGSLGAAFSALLADKAKLTALVGSVTALAAG 256
E.falciformis/1-597  190 GRILQERENIDVHLRELRAKAAEDRKTKLESLQAIFGSTGAAFQHLMKDKTQLTTLVASITALAGG 255
B.bovis/1-567        184 IRIEQERKNFDIHVKMMKERSVEERKTKLESLQITFSSLGTAFSSLLADKQRLTAGVTTLSALALG 249
T.equi/1-568         189 GKIKQERENFDVHVKMMKERAIEERQTKLESLNIIFSSLGAAFSSLLADKERLTTGVTALTALAIG 254
T.parva/1-558        186 GKIKQERENFDIHLKMMKERSVEERKTKLESLNIIFSSLGSGLYSLLSDKQRLTYTVMTLTGLSLG 251
B.microti/1-558      185 ARAYVERENFDINLKMLKERSIEERETKLQSLNIIFSSLGNSFRSLIDDKKRLYTFVGSLSALALG 250
C.muris/1-635        189 GKIQLERENLDIHLQELKLRAEENRKTRIESIQSIFGNLSTITGKLYEDKMKLATLIGGVTLTALG 254
C.parvum/1-627       197 GRIKAERENADIRLGEIKAKAKESRTTHLESIKTIFGGIREMGSSLYQDKSKLTMLVGGLTAMAFG 262

P.falciparum/1-663   257 IYTTKHTTKFIRTYAETKLGKPKLIRETSLWHINKF-----FDIFNFKKNFALIKNFIYPFKNKNN 317
P.vivax/1-665        250 IYTTKHTTRLIRSYVETKLGKPKLIRETSLWHINKF-----FDLFNLKKNLLLMKNILQRRSPK-- 308
T.gondii/1-588       258 VYGARTGAHLAGKYWESRLGKPPLVRETSRWVFS-------KSFFSPLRF----------LRGK-- 304
B.besnoiti/1-577     254 VYGARTGAHLLGRYWESRLGKPPLVRETSRWVFS-------KSFFNPLRF----------IRGK-- 300
E.tenella/1-595      257 IYGARAAASIAARYVESRIGKPPLVRETSRWTFS-------GGPFSLLRP----------WRPR-- 303
E.falciformis/1-597  256 IYGARAAASLGGRYLEARMGKPPLVRETSRWTFT-------RSVFSPLRF----------LRGRK- 303
B.bovis/1-567        250 IYGAKNGTRLAGRILERRLGKPPLVRETSRWTLM-------GGISNLFKR----------YFPT-- 296
T.equi/1-568         255 VYGARAGTRVLGKFMEQKIGKPPLVRDTSRWVLM-------NGLGNFVKG----------LVKT-- 301
T.parva/1-558        252 VYTAKNGTKVARKVIEQKIGKPSLVRETSKSIIT-------NNLRSFWES----------LMGK-- 298
B.microti/1-558      251 VYGARAGTELAKKVFEKRIGKPTLVRETSKWVMM-------NSLRNFLSF----------RYFT-- 297
C.muris/1-635        255 IYGSRSTAQVIAGYFESRLGKPSLVRETSRNKFSYL-----GDFVAKQTSFLKLLTSF--MRKS-- 311
C.parvum/1-627       263 IYGAKNTTRVVANMIETSFGRPSLIRETNMSFLTRHGLKVRNNFFSPAIAF-RLLG----LRNK-- 321

P.falciparum/1-663   318 LYNNYKIFDQIVLNEELQEKLQWSINSLKNSNKYNLYLKNILLHGPPGTGKTLFAKTLSYHSNFDY 383
P.vivax/1-665        309 ---ESNFFTNIVLNEELQEKLSWSINSLTNSKRYDLYLKNILLHGPPGTGKTLFAKTLSHFSNFDY 371
T.gondii/1-588       305 --PKKDFQEKIVLEEELAERLQWTTNSLIASKANGTPFRHMLLYGAPGTGKTLFARTLARESGMDY 368
B.besnoiti/1-577     301 -VQKKDFQEKIVFEEELAERLQWTTNSLISAKANGTPFRHMLLYGAPGTGKTLFARTLARESGMDY 365
E.tenella/1-595      304 --GAPQTKENIVLEKDLFQKLEFSKNSLLTTKAHNSHFRHLLLHGPPGTGKTLFARTLARESGMDY 367
E.falciformis/1-597  304 ASASEQLQERLVLEPELAERLQWTTNSLLTTKAHGVPFRHLLLHGPPGTGKTLFARTLARASGMDY 369
B.bovis/1-567        297 --GNVNALTKIVLDNNLHQRLSWTTNSLMNAKKNGAPFRNLLLYGPPGTGKTLFAKTLASNSGMDF 360
T.equi/1-568         302 --NKELKIDQIVLNDQLYQRLNWTVNSLVRAKENGTNFRHILLYGPPGTGKTLFAKTVAKRSGMDY 365
T.parva/1-558        299 --KKELNLNEIVLNNKLSERLNWSINSLLKCKENKTPYRNILLYGPPGTGKTLFAKTLALKSGMDY 362
B.microti/1-558      298 --KRYPKIDSIVLQPELKQRLEWTTNSLVSAKNNKIPYRHILLYGPPGTGKTLFAKTIAKNSGMDY 361
C.muris/1-635        312 --NTSAICEDIILPKDLQERLEWTVNSLVNSRKNNIPFRHMLLWGAPGTGKTLFARTLALKCGMDY 375
C.parvum/1-627       322 LVKKPKVFENIVLPSELENRLNWTVNTLVNSRRFDVPFRNMLLWGKPGTGKTMFARKLAKESGLDY 387
```

- Outer Membrane Helix
- Bridge
- Inner Membrane Helix
- Walker A
- Walker B
- Arginine Finger

```
P.falciparum/1-663  384 IIINGGDVSALGIHASVELNKIFDFLKRRKNKKCIIFIDEAEAFLRKGRN-----ESSIH------ 438
P.vivax/1-665       372 IIINGGDVSALGVHASVELNKIFDFIKRRKNKKCVIFFDEAEAFLRRGRN-----ESSAH------ 426
T.gondii/1-588      369 AIMTGGDVGPLGMDAPNEINKLFSWANKSR-KGLLLFIDEADAFLRQGR------GTARG------ 421
B.besnoiti/1-577    366 AIMTGGDVGPLGRDGPSEVNKLFAWADKSR-KGLLLFIDEADAFLRQGR------GSASA------ 418
E.tenella/1-595     368 AIMTGGDVGPLGPLGASEINKLFNWAQKSR-KGLILFIDEADAFLRQGRAAAAAAAAAAAPAAAAA 432
E.falciformis/1-597 370 AIMTGGDVGPLGREGAAEMNKLFAWAEKSR-KGLILFIDEADAFLRQGRAAVPGTGGAEG------ 428
B.bovis/1-567       361 AIMTGGDIGPLQEEAASEINKLFKWAKKTK-KGLLLFIDEADAFLRQGR------SSANG------ 413
T.equi/1-568        366 AIMTGGDVGPLREEAASEINRLFEWSKKSK-RGLVLFIDEAEAFLRKGR------SSVQG------ 418
T.parva/1-558       363 AIMTGGDVGPLKEDAVTELNKLFKWSNKSK-KGLILFIDEAEAFLRQGR------STLQG------ 415
B.microti/1-558     362 AIVTGGDIGPLGEEGASEINKLFDWAKNSK-RGLILFIDEADAFLRKGR------AQIGQ------ 414
C.muris/1-635       376 AIMTGGDVGPLGRDAANELNKLFKWAKMSR-HGLILFIDEAEAFLRKGR------ESTDS------ 428
C.parvum/1-627      388 AIMSGGDVGKLGKNGVTELNKVFDWARKSN-KGMLLFIDEAEAFLSKGRE-----STTSS------ 441

P.falciparum/1-663  439 ---FSESLRNALATFLYHTGSESKKYSIILATNCKDILDQAVIDRIDEQYNFHNPNIKEIQKMLTM 501
P.vivax/1-665       427 ---FSESLRNALATFLYHTGTESKKFCIILATNCREILDPAVIDRIDEQYIFDFPKINEIRKMLSL 489
T.gondii/1-588      422 ---MSEDMRNALSAFLHHTGTENDKFCVILATNCREILDRAVLDRVDEQFEFPLPAVEERKRMLKQ 484
B.besnoiti/1-577    419 ---MSEDARNAISAFLHHTGTESDKFCVVLATNCREILDRAVLDRVDEQFEFPLPAVEERKRMLNQ 481
E.tenella/1-595     433 AADMSEHSRNALSAFLHHTGTETNKFCLILATNCKEILDKAVLDRIDEQFEFNLPAAAERYKMLQQ 498
E.falciformis/1-597 429 ---MSEDSRNAVSAFLHHTGTETDKFCVVLATNCREILDRAVLDRVDEQFEFPLPGTPERLRMLCQ 491
B.bovis/1-567       414 ---MSENMRNALSAFLYHTGTESKELSLILATNEREILDKAVLDRMDEQYEFGLPQLEERKRMIAM 476
T.equi/1-568        419 ---MSENVRNALSAFLYHTGTETDKFCLILATNERDILDPAIVDRMDEQYEFPLPETNERKRMITL 481
T.parva/1-558       416 ---MSENIRNALSTFLYHTGNENNNFCLILATNEKDILDKAVVDRIDESYNFDLPEEEERKRMIKI 478
B.microti/1-558     415 ---MSENVRNALSAFLYQTGTETTKFCLILATNEKNILDPAILDRVDEKFNFELPGLEERKMMIKL 477
C.muris/1-635       429 ---ISENMRNVLSSFLYHTGTESKDLCILLATNAPECLDRAILDRVDESFEFPLPKHSERTMMINM 491
C.parvum/1-627      442 ---KSENSRNALSAFLHQTGTESKDICILLATNVPGTLDSAVIDRVDEVFEFPNPGFNERLKLIKQ 504

P.falciparum/1-663  502 YFNKYVYPLK-----------KY-----------NITIDSSIDNEYIHNLSNKLCGLSGRQISKLC 545
P.vivax/1-665       490 YFNKYVFPLK-----------KY-----------NIVVDASIDDLYLDVLASRLVGLSGRQISKLC 533
T.gondii/1-588      485 FLDEYIHRTT-----------PTGR---------KIVVDENIDDAFVCEMAEKTEGFSGRQLAKLV 530
B.besnoiti/1-577    482 FLEEYIFRTT-----------KTGR---------KIVVDEKIDDAFVQEMAEKTEGFSGRQLAKLV 527
E.tenella/1-595     499 FMDRYIRSRSSSSSSSGSSGKSSSN---------SIVVDERINDEFLKDVADKTEGFSGRQLAKLV 555
E.falciformis/1-597 492 FIDRYLKREG-EPTKQA----ETGK---------KVVIDQEINDEFMQEMAEKTEGFSGRQLAKLV 543
B.bovis/1-567       477 FMKKYVLTPT-----------TRGN---------KVEIDENINDDFFAKVAERTEGFSGRQLSKMC 522
T.equi/1-568        482 FMHQFVINPT-----------KRGK---------KIQIDPRINDEFYAKVAEKTEKLSGRQLAKLC 527
T.parva/1-558       479 FMYQYVINPL-----------KRTS---------KVQIDEGINDQYFAKLAKKTQGLSGRQISKLC 524
B.microti/1-558     478 FMEQYVIGPS-----------KNDK---------TIVIDPKINESFNDKVARNTQGFSGRQLAKFC 523
C.muris/1-635       492 FLNRNFPQNS-----------VRSKRY-------NIRLDPAIDTTFVDYLASRTEGFSGRQLSKLI 539
C.parvum/1-627      505 FLELNFNCSY-----------ESGKFINLPSLYNSIKIHPSLDQTFLDVLARKTEGFSGRQLFKLV 559

P.falciparum/1-663  546 LNIQSCVFGSDTKVVTKELINLITAWHLSNSLE-----QTNNQNV---------------NKKKQH 591
P.vivax/1-665       534 LNIQNCVFGSNSKVVSKDLIDLIVSWNLSNSFETRGEMQTSRQHVAAPIPRGSASGISPGATKEGD 599
T.gondii/1-588      531 IAFQAAVFGSGTNTLTRGMAETVLSWKLAHFDQDI-------------------------DTVERR 571
B.besnoiti/1-577    528 IAFQAAVFGSGTNTLTRGMAETVLSWKLAHFDQDI-------------------------DTIERR 568
E.tenella/1-595     556 LAMQAAAYGSGTNALTLGLAEAVLKWRLKSHNEEQ-------------------------QKQQH- 595
E.falciformis/1-597 544 LAMQAAVFGSGTNTLTRGMAAAVLDWKVKHLTEDV-------------------------EMLDRE 584
B.bovis/1-567       523 IAIQSAVFGSGTTRLSLELAETVINWHIDEHRKNH-------------------------KTTEHA 563
T.equi/1-568        528 ISLQSAVYGSGTTQLTLELANTVIDWHLERFNKGI-------------------------ADKDDI 568
T.parva/1-558       525 ISLQSAIYGSGASKLTVDLADTVIDWHLKNQNND-------------------------------- 558
B.microti/1-558     524 ISLQSALFGSGSKILSVDLAESILNWHLSQEKNLA------------------------------- 558
C.muris/1-635       540 IGMQAAALGSGSNILTKGLAEAVLVWKLAYKDELKT--NFGSNIYNS------------DGVMDIH 591
C.parvum/1-627      560 LGMKSIVLGSGVESLTREIAESALSWKLDGENNLNL--NSSYSSTS--------------SKSSAN 609

P.falciparum/1-663  592 SSNYTSSDDNS--NFKLKDNPNVHKKKNEQHHNITNIEQEHNKKENDENSKNINLN--NTPNHDA- 652
P.vivax/1-665       600 SNAGTNSGENSKPDMGLGVGANVGSNLAGQPSTVAASMARSVAGLSGDDSKE---E--NSP----- 655
T.gondii/1-588      572 SREQKLSGAQ------------------------------------TEASTQI------------- 588
B.besnoiti/1-577    569 SREQKLADA--------------------------------------------------------- 577
E.tenella/1-595         ------------------------------------------------------------------
E.falciformis/1-597 585 KRHMKLKNAN-------------------------------------------------------- 594
B.bovis/1-567       564 EPAD-------------------------------------------------------------- 567
T.equi/1-568            ------------------------------------------------------------------
T.parva/1-558           ------------------------------------------------------------------
B.microti/1-558         ------------------------------------------------------------------
C.muris/1-635       592 KKSVIQYNHNY----------------------------------PKEDTGRLALNIGTSTNSSSK 623
C.parvum/1-627      610 QSSHINCDNS------------------------------------NSDLTNLE------------ 627

P.falciparum/1-663  653 -IKKKVLINEQL 663
P.vivax/1-665       656 --QVKTKVGMQH 665
T.gondii/1-588          ------------
B.besnoiti/1-577        ------------
E.tenella/1-595         ------------
E.falciformis/1-597 595 ---------LKE 597
B.bovis/1-567           ------------
T.equi/1-568            ------------
T.parva/1-558           ------------
B.microti/1-558         ------------
C.muris/1-635       624 SDDQVQISRVTA 635
C.parvum/1-627          ------------
```

Outer Membrane Helix

Bridge

Inner Membrane Helix

Walker A

Walker B

Arginine Finger

S1 Fig. (A) Confidence and similarity metrics for structural assembly modelling. **(B)** Phylogenetic analyses showing the evolutionary relationship between homologs of ATAD3 amongst alveolates and other eukaryotes including *Homo sapiens*, *Arabidopsis thaliana, Caenorhabditis elegans, Plasmodium falciparum, Toxoplasma gondii,* and *Cryptosporidium parvum*. AFG3-like proteins were chosen for the putative outgroup as selected members of the AFG3-like proteins exhibited the highest similarity to *Pf*ATAD3 and this analysis is limited to the NTPase domain because no other AAA+ protein subclass has detectable similarity with the ATAD3 domain. **(C)** Phylogenetic analyses showing the evolutionary relationship between homologs of ATAD3 amongst alveolates and other eukaryotes including *Homo sapiens*, *Arabidopsis thaliana, Caenorhabditis elegans, Plasmodium falciparum, Toxoplasma gondii,* and *Cryptosporidium parvum* using the full-length ATAD3 sequence. **(D)** Multiple sequence analysis of ATAD3 homologs in apicomplexan parasites.
