# Supplementary material for: ATAD3 megadalton complex in Plasmodium falciparum is essential for mitochondrial and cellular viability
Source: PLoS Pathog. 2026 Jun 3;22(6):e1014317. doi: 10.1371/journal.ppat.1014317 (PMC13249166; doi:10.1371/journal.ppat.1014317)
Supplement: S2 Fig — The sequences are color coded and identified in a key below the synthetic construct. (B) CRISPR-Cas9 homologous recombination integration cloning strategies to generate endogenously tagged PfATAD3-HA-TetR, PfATAD3-HA-TetR/TOM22-mNG and PfATAD3-HA-TetR/mito-mScarlet transgenic parasite lines using corresponding parental lines. (C) Plasmid map of the pMGBKRML-PfATAD3-HA plasmid for endogenous tagging and conditional expression. Restriction sites and the identity of each region are indicated. Created with SnapGene. (D) Plasmid map of the pMKCas9 plasmid for guide RNA construction. Restriction sites and the identity of each region are indicated. Created with SnapGene. (E) Integration PCR confirming presence of HA-tagged ATAD3 construct with TetR aptamers in both PfATAD3-HA-TetR/TOM22-mNG and PfATAD3-HA-TetR/mito-mScarlet transgenic parasite lines. Band sizes are denoted in blue while marker sizes are in black. (PDF) [file ppat.1014317.s002.pdf]

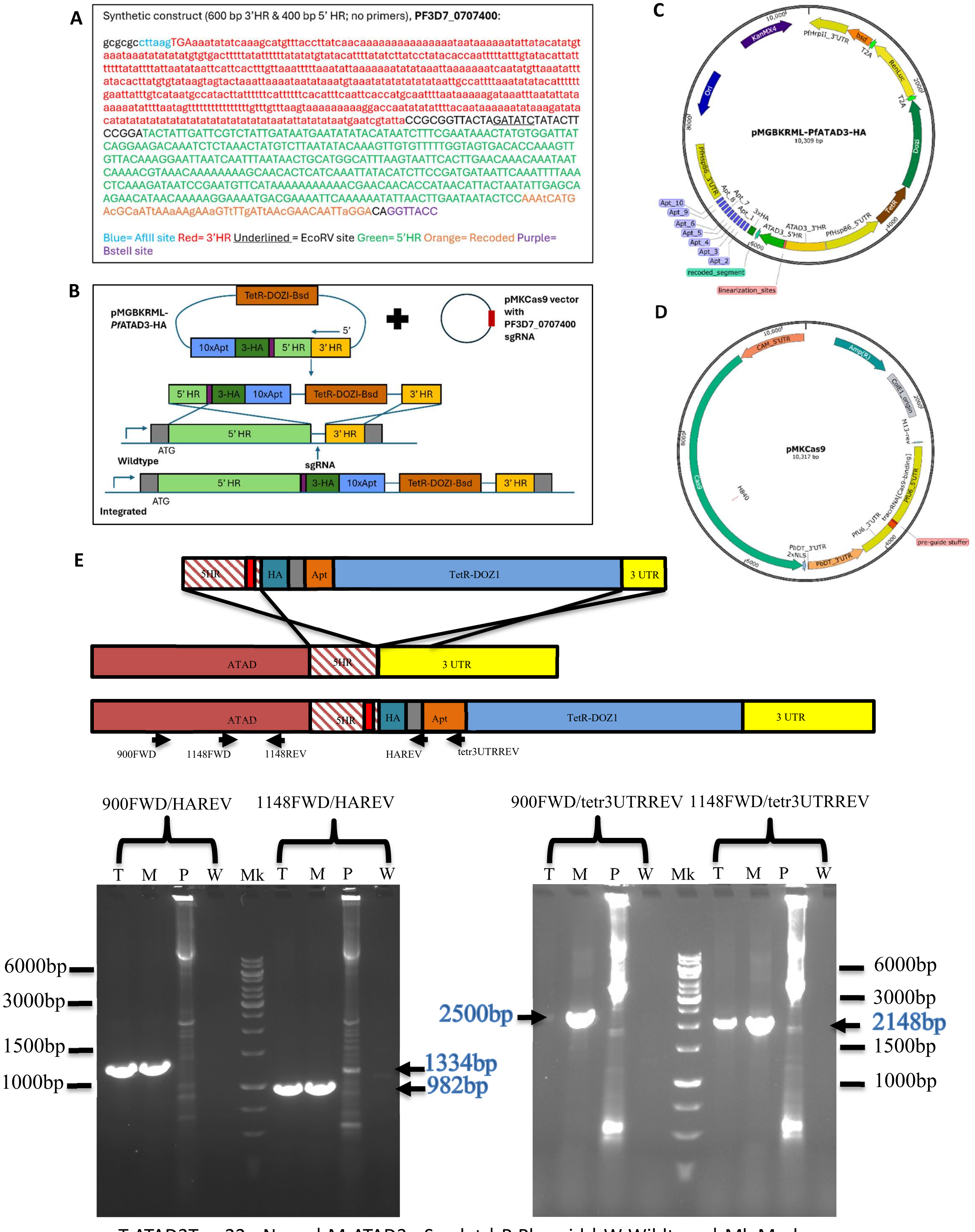

A
Synthetic construct (600 bp 3'HR & 400 bp 5' HR; no primers), PF3D7_0707400:
gcgcgccttaagTGAaaatatatcaaagcatgtttaccttatcaacaaaaaaaaaaaaaaataataaaaaatattatacatatgt
aaataaatatatatatgtgtgactttttatatttttatatatgtatacattttatatcttatcctatacaccaatttttatttgtatacattatt
tttttatattttattaatataattcattcactttgttaaatttttaaatattaaaaaaatatataaattaaaaaaatcaatatgttaaatattt
atacacttatgtgtataagtagtactaaattaaaataatataaatgtaaatatatatatatataattgccattttaaatatatacatttttt
gaattatttgtcataatgccatacttatttttcatttttcacatttcaattcaccatgcaattttaataaaaagataaatttaatattata
aaaaatatttaatagttttttttttttttttgtttgtttaagtaaaaaaaaaggaccaatatatattttacaataaaaaatataaagatata
catatatatatatatatatatatatatatatataatattatatataatgaatcgtattaCCGCGGTTACTAGATATCTATACTT
CCGGATACTATTGATTCGTCTATTGATAATGAATATATACATAATCTTTCGAATAAACTATGTGGATTAT
CAGGAAGACAAATCTCTAAACTATGTCTTAATATACAAAGTTGTGTTTTTGGTAGTGACACCAAAGTT
GTTACAAAGGAATTAATCAATTTAATAACTGCATGGCATTTAAGTAATTCACTTGAACAAACAAATAAT
CAAAACGTAAACAAAAAAAAGCAACACTCATCAAATTATACATCTTCCGATGATAATTCAAATTTTAAA
CTCAAAGATAATCCGAATGTTCATAAAAAAAAAAAACGAACAACACCATAACATTACTAATATTGAGCA
AGAACATAACAAAAAGGAAAATGACGAAAATTCAAAAAATATTAACTTGAATAATACTCCAAAtCATG
AcGCaATtAAaAAgAAaGTtTTgATtAAcGAACAATTaGGACAGGTTACC
Blue= AflII site Red= 3'HR Underlined = EcoRV site Green= 5'HR Orange= Recoded Purple= Bstell site
B
TetR-DOZI-Bsd
pMGBKRML-PfATAD3-HA
5'
10xApt
3-HA
5' HR
3' HR
pMKCas9 vector with PF3D7_0707400 sgRNA
5' HR
3-HA
10xApt
TetR-DOZI-Bsd
3' HR
ATG
Wildtype
sgRNA
Integrated
C
pMGBKRML-PfATAD3-HA
10,309 bp
KanMX4
PfHrpII_3'UTR
bsd
T2A
RenLuc
Ori
DozI
TetR
PfHsp86_5'UTR
PfHsp86_3'UTR
ATAD3_3'HR
ATAD3_5'HR
3xHA
Apt_1
Apt_2
Apt_3
Apt_4
Apt_5
Apt_6
Apt_7
Apt_8
Apt_9
Apt_10
recoded_segment
linearization_sites
D
pMKCas9
10,317 bp
CAM_5'UTR
Amp(R)
ColE1_origin
M13-rev
Cas9
H840
PfU6_5'UTR
tracrRNA[Cas9-binding]
PfU6_3'UTR
pre-guide stuffer
PbDT_3'UTR
2xNLS
E
5HR
HA
Apt
TetR-DOZ1
3 UTR
ATAD
900FWD
1148FWD
1148REV
HAREV
tetr3UTRREV
900FWD/HAREV
1148FWD/HAREV
900FWD/tetr3UTRREV
1148FWD/tetr3UTRREV
T M P W Mk T M P W
T M P W Mk T M P W
6000bp
3000bp
1500bp
1000bp
1334bp
982bp
2500bp
2148bp
T-ATAD3Tom22mNeon | M-ATAD3mScarlet | P-Plasmid | W-Wildtype | Mk-Marker

S2 Fig. (A) Sequence of synthetic gene used to create the endogenously tagged *Pf*ATAD3A parasite line. The sequences are color coded and identified in a key below the synthetic construct. **(B)** CRISPR-Cas9 homologous recombination integration cloning strategies to generate endogenously tagged *Pf*ATAD3-HA-TetR, *Pf*ATAD3-HA-TetR/TOM22-mNG and *Pf*ATAD3-HA-TetR/mito-mScarlet transgenic parasite lines using corresponding parental lines. **(C)** Plasmid map of the pMGBKRML-*Pf*ATAD3-HA plasmid for endogenous tagging and conditional expression. Restriction sites and the identity of each region are indicated. Created with SnapGene. **(D)** Plasmid map of the pMKCas9 plasmid for guide RNA construction. Restriction sites and the identity of each region are indicated. Created with SnapGene. **(E)** Integration PCR confirming presence of HA-tagged ATAD3 construct with TetR aptamers in both *Pf*ATAD3-HA-TetR/TOM22-mNG and *Pf*ATAD3-HA-TetR/mito-mScarlet transgenic parasite lines. Band sizes are denoted in blue while marker sizes are in black.
