# Supplementary material for: ATAD3 megadalton complex in Plasmodium falciparum is essential for mitochondrial and cellular viability
Source: PLoS Pathog. 2026 Jun 3;22(6):e1014317. doi: 10.1371/journal.ppat.1014317 (PMC13249166; doi:10.1371/journal.ppat.1014317)
Supplement: S3 Fig — (B) Immunofluorescence Assay (IFA) demonstrating colocalization of PfATAD3 to PfTOM22-mNeonGreen (Translocator of Outer Mitochondrial Membrane 22) in the mitochondria of asexual P. falciparum parasites. (PDF) [file ppat.1014317.s003.pdf]

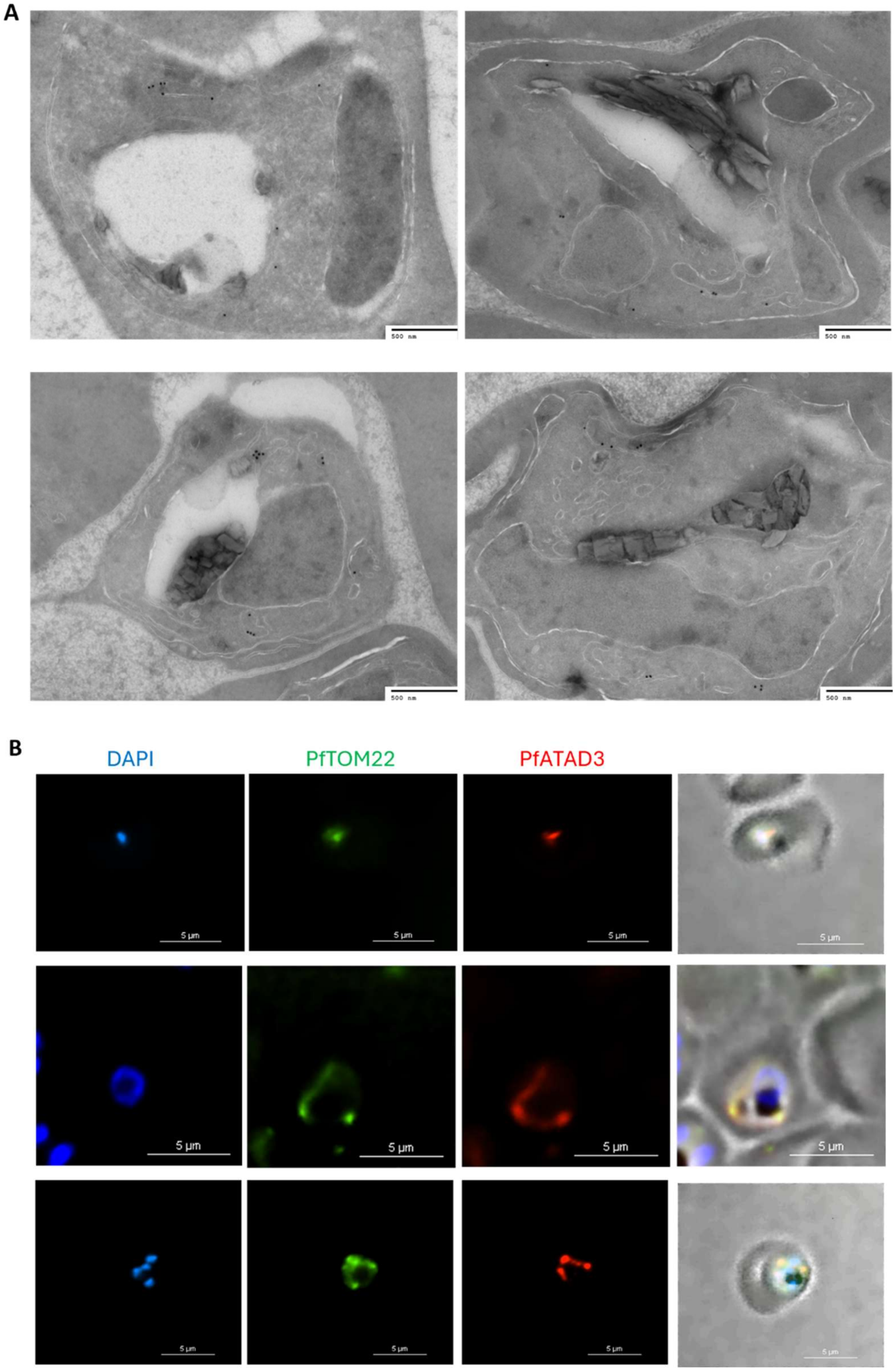

S3 Fig. (A) Immuno-electron Micrographs showing localization of *Pf*ATAD3 to mitochondria of asexual parasites via mouse anti-HA or rabbit anti-HA primary antibodies and corresponding anti-mouse or anti-rabbit 18 nm colloidal gold particles. **(B)** Immunofluorescence Assay (IFA) demonstrating colocalization of *Pf*ATAD3 to *Pf*TOM22-mNeonGreen (Translocator of Outer Mitochondrial Membrane 22) in the mitochondria of asexual *P. falciparum* parasites.
