# Supplementary figures and images for: ATAD3 megadalton complex in Plasmodium falciparum is essential for mitochondrial and cellular viability
Source: PLoS Pathog. 2026 Jun 3;22(6):e1014317. doi: 10.1371/journal.ppat.1014317 (PMC13249166; doi:10.1371/journal.ppat.1014317)

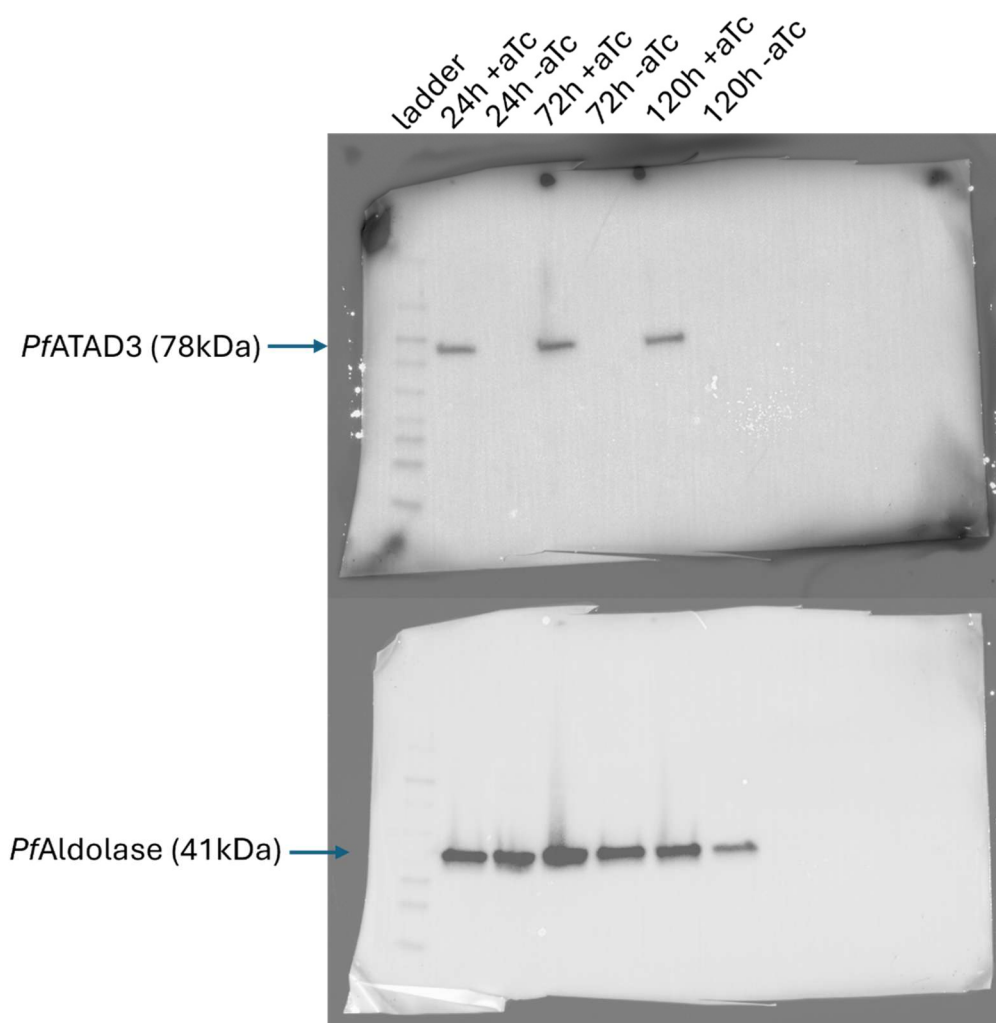

**S4 Fig.** Full gel image representative of the growth assay western blot.

Supplement: S4 Fig — (PDF) [file ppat.1014317.s004.pdf]
