# Supplementary material for: ATAD3 megadalton complex in Plasmodium falciparum is essential for mitochondrial and cellular viability
Source: PLoS Pathog. 2026 Jun 3;22(6):e1014317. doi: 10.1371/journal.ppat.1014317 (PMC13249166; doi:10.1371/journal.ppat.1014317)
Supplement: S5 Fig — Scale bar is 10 µm (C) An enlarged view of a representative cell from (B). (D) Representative full northern blot (left) and denaturing agarose RNA gel (right) demonstrating reduction of processed mitochondrial RNA transcripts upon knockdown of PfATAD3. (PDF) [file ppat.1014317.s005.pdf]

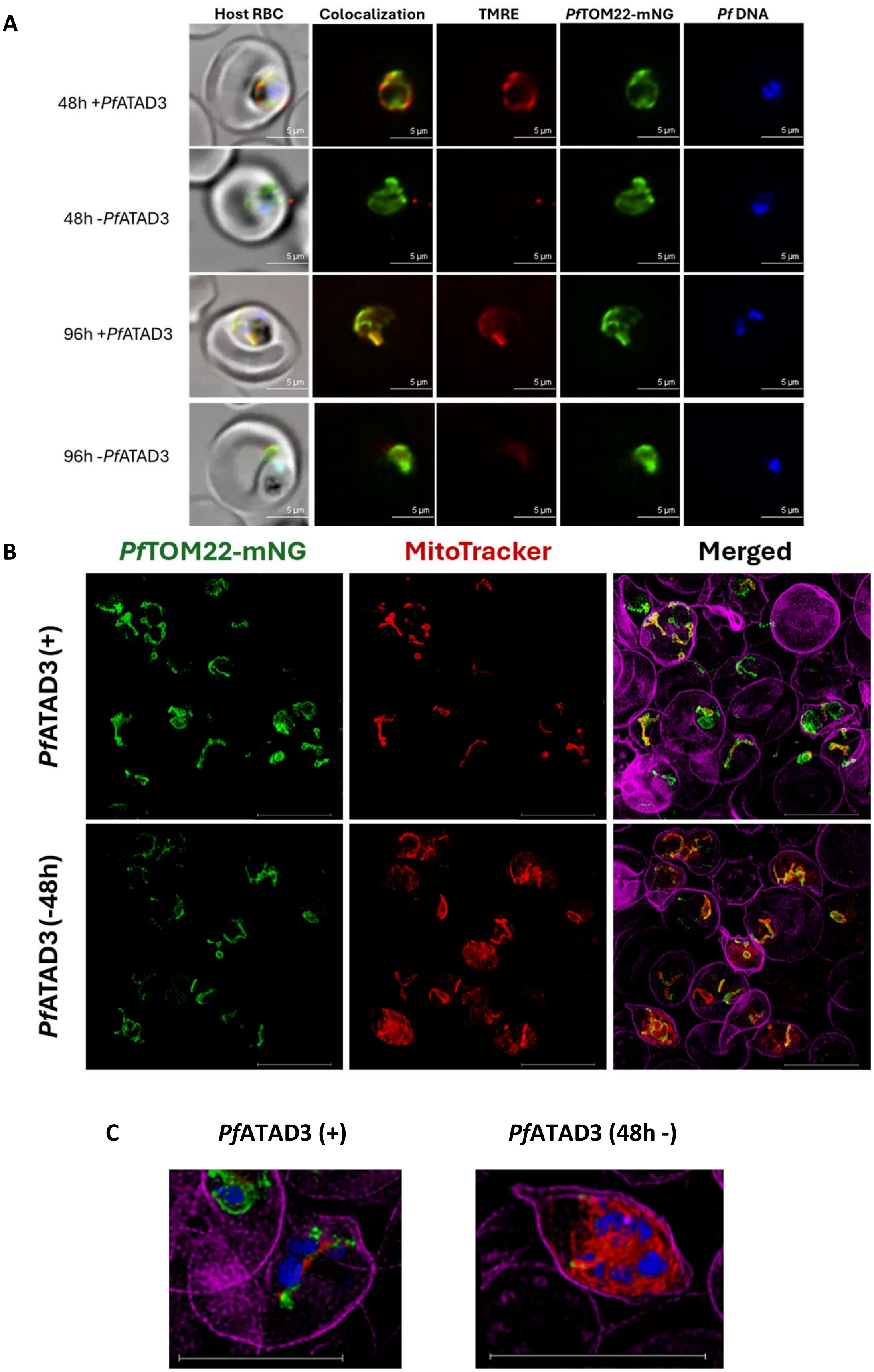

A
Host RBC
Colocalization
TMRE
PfTOM22-mNG
Pf DNA
48h +PfATAD3
48h -PfATAD3
96h +PfATAD3
96h -PfATAD3
5 µm
B
PfTOM22-mNG
MitoTracker
Merged
PfATAD3(+)
PfATAD3(-48h)
C
PfATAD3 (+)
PfATAD3 (48h -)

D

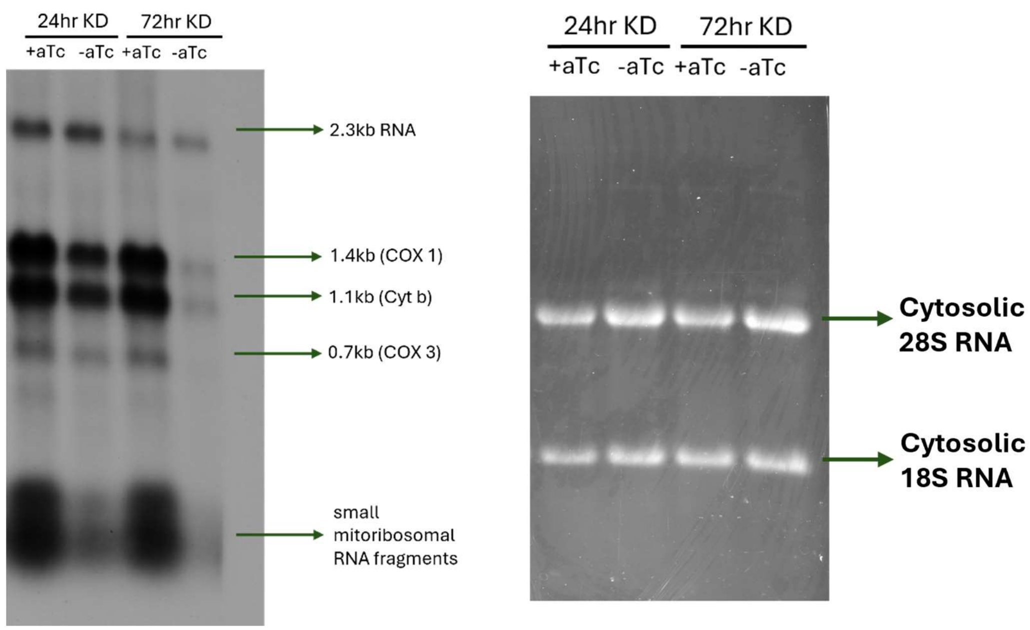

S5 Fig. (A) Live cell imaging of +/-*Pf*ATAD3 (48h and 96h) TetR/TOM22-mNG parasites stained with TMRE dye to assess mitochondrial membrane potential upon loss of *Pf*ATAD3 **(B)** Live Cell Scanning Confocal Super-resolution Microscopy of +/-*Pf*ATAD3 (48h) TetR/TOM22-mNG parasites [*Pf*DNA – DAPI (Blue); Outer mitochondrial membrane – *Pf*TOM22-mNG (Green); Mitochondrion – MitoTracker (Red); Host red blood cell – Wheat Germ Agglutinin (Purple). Scale bar is 10 µm **(C)** An enlarged view of a representative cell from **(B). (D)** Representative full northern blot (left) and denaturing agarose RNA gel (right) demonstrating reduction of processed mitochondrial RNA transcripts upon knockdown of *Pf*ATAD3.
