# Supplementary material for: ATAD3 megadalton complex in Plasmodium falciparum is essential for mitochondrial and cellular viability
Source: PLoS Pathog. 2026 Jun 3;22(6):e1014317. doi: 10.1371/journal.ppat.1014317 (PMC13249166; doi:10.1371/journal.ppat.1014317)
Supplement: S6 Fig — PfEXP2 was used as a loading control. (B) Full large pore composite native gel showing PfATAD3 is present in a mega-Dalton hetero-oligomeric complex. (C) Large pore composite native gel showing aTc washout for 24h induces knockdown of the giant megaDalton PfATAD3 complex. (PDF) [file ppat.1014317.s006.pdf]

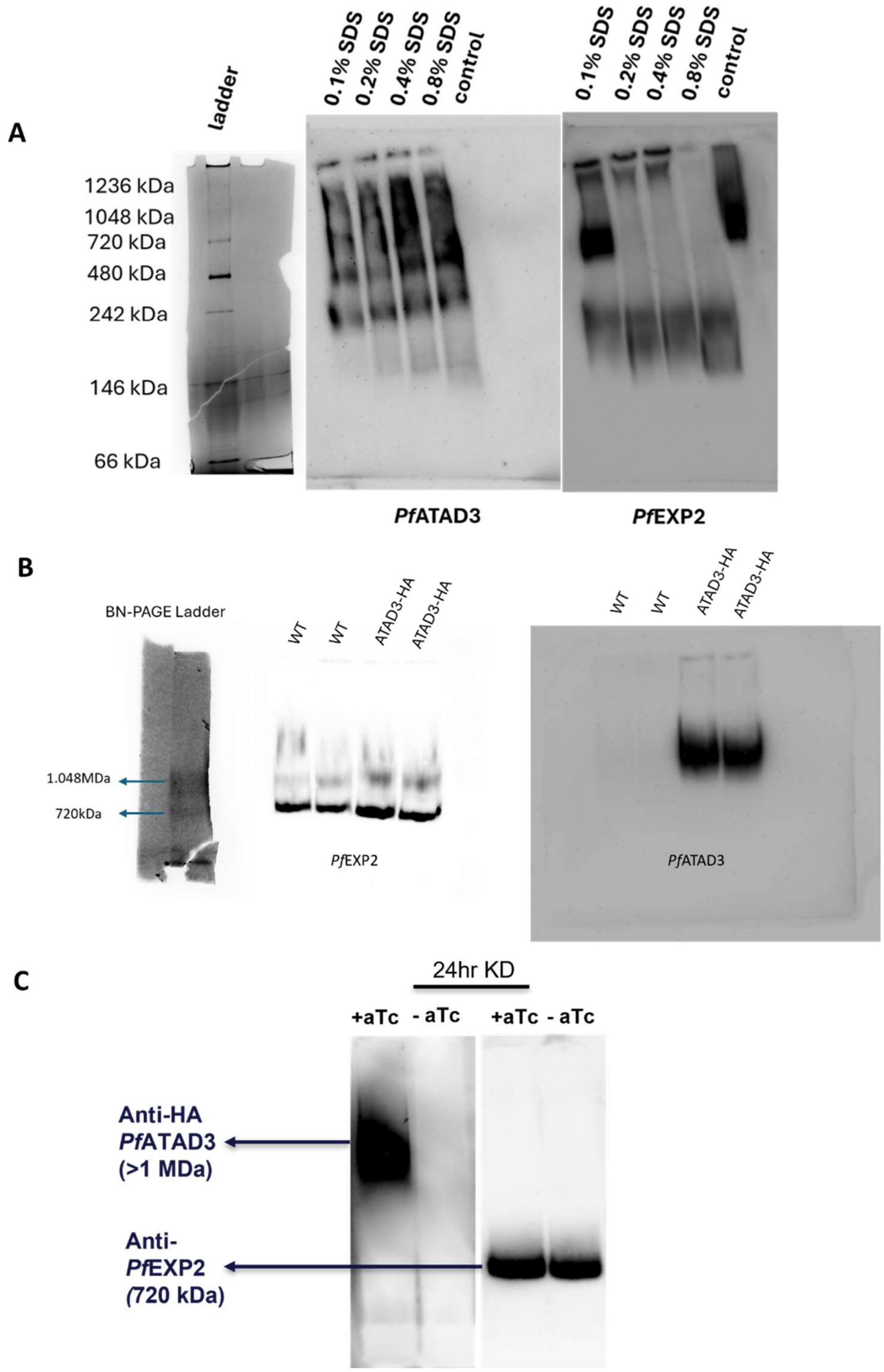

S6 Fig. (A) Blue-Native PAGE of control and SDS-treated solubilized extract demonstrating the presence of multiple sub-complexes of *Pf*ATAD3. *Pf*EXP2 was used as a loading control. **(B)** Full large pore composite native gel showing *Pf*ATAD3 is present in a mega-Dalton hetero-oligomeric complex. **(C)** Large pore composite native gel showing aTc washout for 24h induces knockdown of the giant megaDalton *Pf*ATAD3 complex.
