# Supplementary material for: ATAD3 megadalton complex in Plasmodium falciparum is essential for mitochondrial and cellular viability
Source: PLoS Pathog. 2026 Jun 3;22(6):e1014317. doi: 10.1371/journal.ppat.1014317 (PMC13249166; doi:10.1371/journal.ppat.1014317)
Supplement: S1 Table — (PDF) [file ppat.1014317.s007.pdf]

S1 Table. Enriched Interacting Protein Partners of *Pf*ATAD3-HA in asexual *P. falciparum* parasites.

Gene	Protein Name	Fold Change	P-value	MIS	Prior Localization Information
PF3D7_1102700	early transcribed membrane protein 11.1	9.43E+08	0.018038	1	Symbiont-containing vacuole membrane
PF3D7_0707400	ATPase family AAA domain-containing protein 3A, putative	7.38E+08	0.010399	0.13	Mitochondrion
PF3D7_1469200	shewanella-like protein phosphatase 1, putative	72738111	0.034519	0.162	Nucleus, Cytoplasm, Endoplasmic Reticulum
PF3D7_1330600	elongation factor Tu, putative	51627111	0.000307	0.131	Mitochondrion, Plastid, Nucleus
PF3D7_1119600	ATP-dependent zinc metalloprotease FTSH	48661111	0.008991	0.141	Mitochondrial Inner Membrane
PF3D7_0726900	mitochondrial import inner membrane translocase, TIM50	43172111	0.009813	0.12	Mitochondrial Inner Membrane
PF3D7_1430200	plasmepsin IX	42511111	0.000571	0.238	Rhoptry
PF3D7_1356200	mitochondrial import inner membrane translocase, TIM23	38442111	0.013472	0.12	Mitochondrial Inner Membrane
PF3D7_0212900	arginyl-tRNA--protein transferase	34974111	0.032805	0.999	Cytoplasm
PF3D7_0413500	phosphoglucomutase-2	34916111	0.027304	0.119	Cytoplasm, Mitochondrion Membrane
PF3D7_0419600	ran-specific GTPase-activating protein 1, putative	34629111	0.045771	0.998	Cytoplasm, Nuclear Pore, Nucleus
PF3D7_1468100	MORC family protein	31152111	0.012493	0.179	Nucleus
PF3D7_1462300	GTP-binding protein, putative	29693111	0.013979	0.178	Nucleus
PF3D7_1104100	syntaxin, Qa-SNARE family	24921111	0.009559	0.643	Vesicle Membrane, Food vacuole, Plasma Membrane
PF3D7_0305600	DNA-(apurinic or apyrimidinic site) endonuclease	21177111	0.002241	0.166	Mitochondrion, Nucleus
PF3D7_0303000	N-ethylmaleimide-sensitive fusion protein	16594111	0.020639	0.186	Golgi stack, Food vacuole, Host cell cytoplasm
PF3D7_0408500	flap endonuclease 1	15149111	0.004239	0.999	Mitochondrion, Nucleolus, Nucleoplasm
PF3D7_0207500	serine repeat antigen 6	8528411	0.010314	0.252	Symbiont-containing vacuole membrane
PF3D7_0320800	ATP-dependent RNA helicase DDX6	19.19762	0.004638	1	P-body, Cytoplasm, Cytoplasmic stress granule, Nucleus
PF3D7_1434800	Mitochondrial acidic protein MAM33, putative	11.78068	0.017136	0.509	Mitochondrial Matrix
PF3D7_0617900	histone H3 variant	8.421096	0.041978	0.411	Nucleosome
PF3D7_0610400	histone H3	5.103586	0.040886	0.176	Nucleosome
PF3D7_1441100	conserved Plasmodium protein, unknown function	4.188605	0.04055	1	Nucleus
