# Supplementary material for: ATAD3 megadalton complex in Plasmodium falciparum is essential for mitochondrial and cellular viability
Source: PLoS Pathog. 2026 Jun 3;22(6):e1014317. doi: 10.1371/journal.ppat.1014317 (PMC13249166; doi:10.1371/journal.ppat.1014317)
Supplement: S2 Table — (PDF) [file ppat.1014317.s008.pdf]

S2 Table. Primer sequences used in parasite line generation and validation

Primer Name	Sequence
0707400 FWD	GATGTACCTAGGtaaaATGAATTTTCCTAATTTGAGTAAGAAAATAAATTC
0707400 REV	GATGTACGTACGgttCAATTGTTCATTTATTAATACCTTTTTCTTTATAGC
PfTom22 FWD	cgTGTACAAGggatctggatctCGTACGATGGGAACAGCACTATCAAAAATTATTACG
PfTom22 REV	gtCTTAAGTTAGTTTAATTGTGGAACATTGGC
PF3D7_0707400gRNA1	CATATTAAGTATATAATATTGTTCTTTATAGCATCATGGTTGTTTCAGAGCTATGCTGGA
PF3D7_0707400gRNA2	atttCATATTAAGTATATAATATTGCCATGATGCTATAAAGAAAAGTTTCAGAGCTATGCTGGAaac
PF3D7 6kb 4316 REV	GGTttaCTTggaacagtttttAACattgc
PF3D7 6kb 4373 FWD	GCAgaaCAAagaagtttaacaactataattc
ATAD3-1148 FWD	TTATTAACGGAGGTGATGTTAGTG
ATAD3-900 FWD	AGAACATATGCAGAAACAAAATTAGG
HA-REV	GGCCCGAATTCTCATCATTGTGC
3'TetR-UTR-check	ATATTTCATGTCTCAGTAAAGTCTTTCAATAC
