# Supplementary material for: ATAD3 megadalton complex in Plasmodium falciparum is essential for mitochondrial and cellular viability
Source: PLoS Pathog. 2026 Jun 3;22(6):e1014317. doi: 10.1371/journal.ppat.1014317 (PMC13249166; doi:10.1371/journal.ppat.1014317)
Supplement: S3 Table — (PDF) [file ppat.1014317.s009.pdf]

S3 Table. Digital PCR Custom Designed TaqMan Gene Expression Assay Details

Assay ID	Assay Name	Assay Mix Concentration	Reporter 1 Dye	Reporter 1 Concentration (µM)	Reporter 1 Quencher	Forward Primer Concentration (µM)	Reverse Primer Concentration (µM)	Forward Primer Sequence	Reverse Primer Sequence	Reporter 1 Sequence	Context Sequence
AP33C2U	CYTOCHROME_B	20x	FAM	5	NFQ	18	18	TGTACTACATTTTATCTTACCATTTATTGGATTATGTATTGT	GGGTATTTTTAATGCTGTATCATACCCT	CATGGTAGCACAAATC	TGTACTACATTTTATCTTACCATTT
AP7DY7N	GAPDH	20x	VIC	5	NFQ	18	18	TGGTCAATTTCCATGTGAGGTAACC	GATCCTTTTCAGCAAAAACACTGACT	CACGCTGATGGATTTT	TGGTCAATTTCCATGTGAGGTAACC
